# Case and Management Considerations of Low-Grade Cystic Duct Stump Dysplasia after Laparoscopic Cholecystectomy

**DOI:** 10.1155/2024/6682520

**Published:** 2024-03-07

**Authors:** Claire Ufongene, Saran Kunaprayoon, Juan Mestre

**Affiliations:** ^1^Icahn School of Medicine at Mount Sinai, New York, USA; ^2^Mount Sinai Hospital, New York, USA; ^3^Elmhurst Hospital Center, Borough of Queens, New York, USA

## Abstract

Cholecystectomies have become one of the more commonly practiced procedures. As a result, there has been a rise in neoplastic changes in excised specimens. Due to this, surgeons must be prepared to manage possible malignancy after resecting what was previously thought to be a benign gallbladder. While management for high-grade dysplasia has been more clearly laid out in literature, data on management of low-grade dysplasia are limited. Here, we report a novel case of a 46-year-old woman with an incidental low-grade dysplasia of the cystic duct stump after a laparoscopic cholecystectomy for biliary colic. The decision was made to excise the remaining stump without further surveillance postoperatively given benign pathology findings. More documented cases and their management and ultimately longitudinal cohort studies will help facilitate the creation of guidelines for managing this particular pathology.

## 1. Introduction

Cholecystectomies have become one of the more commonly practiced procedures, with more than 1.2 million performed annually in the United States as of 2022 [1]. In turn, we have also seen a rise in incidental gallbladder findings, especially neoplasms, when surgically excising what had been perceived as benign disease [2]. Of neoplasms discovered during routine cholecystectomies, one study found that invasive malignancy made up 56% of incidental neoplasms while low- and high-grade dysplasia accounted for 42% of cases [2]. Although dysplasia makes up almost half of incidental neoplastic changes, there is a paucity of literature defining cases and treatment protocol in these instances. Specifically, reported cases and management of isolated incidental low-grade dysplasia are scarce in the literature. This is of concern as these are premalignant findings. As such, it is critical to understand the best evidence-based treatment protocols in order to optimize patient outcomes. Here, we report a case of a 46-year-old woman with an incidental low-grade dysplasia of the cystic duct stump after a laparoscopic cholecystectomy for biliary colic. To date, only a case series and case report have noted isolated low-grade cystic duct dysplasia on resected margins [2, 3]. This case report overviews the subject and management.

## 2. Case Presentation

M.C. is a 46-year-old woman with past medical history significant for hypothyroidism and hypertension who presented to the emergency department with biliary colic symptoms. The patient was discharged home and later returned for an elective laparoscopic cholecystectomy. Intraoperatively, the patient was found to have cholelithiasis with choledocholithiasis on cholangiogram, which were removed intraoperatively during choledochoscopy. She was discharged on postoperative day one without complications.

Microscopic examination of the gallbladder and cystic duct revealed multiple foci of low-grade dysplasia of the gallbladder and with focal presence on the cystic duct margin. This was characterized by overlapping nuclei, nuclear elongation, hyperchromasia, and increased nuclear to cytoplasmic ratio.

Due to the positive margins and a rarity of such cases, a multidisciplinary team decided it was best clinical practice to proceed with a laparoscopic cystic duct stump reexcision. The second pathology report described focal reactive epithelial atypia with no dysplasia present. Again, there was no sign of malignant invasion, and the patient appeared well clinically. Thus, the patient was discharged on postoperative day one and returned to the clinic two weeks later for routine follow-up without complaints.

## 3. Methods

Literature review on cystic duct stump dysplasia was performed through a PubMed search with dates from 2011 to 2023. Key words included cystic duct stump dysplasia and low-grade and high-grade cystic duct carcinomas. Literature regarding gallbladder carcinoma and cystic duct carcinoma were also reviewed.

## 4. Discussion

### 4.1. Incidence and Presentation

While the gallbladder and cases of high-grade cystic duct dysplasia have been reported and studied in the literature, *low-grade cystic duct dysplasia* is rarely reported. The relevance of low-grade cystic duct dysplasia, specifically at the margin, is twofold. Firstly, the concern is that these may evolve into malignancy, warranting prophylactic excision and/or surveillance. Secondly, low-grade dysplasia at the margin may be an indication of preexisting malignancy that needs further staging. Given these possibilities, it is crucial for the field to better delineate guidelines for the management of such a finding.

Cystic duct cancer (CDC) is a type of gallbladder cancer, according to the American Joint Committee on Cancer Staging Manual, that is rare; only 2.6-3.3% of biliary tumors are primary cystic duct carcinomas. Farrar's [4] criteria published in 1951 defined CDC as a malignancy that was strictly limited to the cystic duct without any other biliary involvement. Since then, many types of classifications have been published including Ozden et al. [5], Kim et al. [6], Yokoyama et al. [7], Nakata et al. [8], and, most recently, Nan et al. [9]. More recent classifications of CDC strive to better differentiate subtypes that invade adjacent structures such as the gallbladder, bile ducts, and more porta hepatis, since CDC often present in advanced stages [9, 10].

### 4.2. Management

As previously mentioned, literature specificizing evidence-based practices for the management of low-grade dysplasia of the cystic duct remains very limited. Our case represents one of the only recorded cases of low-grade dysplasia of the cystic duct margin. While cases pertaining to the cystic duct specifically are rarer, we can look at literature from dysplasia of the gallbladder itself for guidance of management. In the case of high-grade dysplasia of the gallbladder, cholecystectomy is usually adequate treatment as it is rare for the dysplasia to reach the resection margin [3]. One study states that 18.9% of patients with gallbladder dysplasia had carcinoma elsewhere in the pancreatobiliary tree [3]—6 of these cases were low-grade dysplasia of the gallbladder itself. However, cystic duct dysplasia in isolation of the gallbladder neoplasm is quite rare, as only 5 cases were identified out of 193 in a retrospective observational study [6]. Of those 5, one had a cholangiocarcinoma upon further workup. Thus, low-grade dysplasia of the gallbladder, while benign, can be an indicator of the field effect where a neoplastic process is found elsewhere.

Another important consideration is pursuing a biopsy of the cystic lymph node during the index cholecystectomy, which is often overlooked when concern for malignancy is low. Based on the American Joint Committee on Cancer (AJCC) 7th edition manual, cystic, pericholedochal, and posterosuperior peripancreatic nodes are key to staging gallbladder cancer and can guide management of such pathology [12]. If cystic and pericholedochal nodes are positive, then complete dissection of pN1 is required. If posterosuperior peripancreatic nodes are positive, then pN2 dissection should be attempted with or without pancreatoduodenectomy [13]. However, given the 8th edition AJCC manual, the location of the lymph nodes is thought to be less significant than the number of lymph nodes involved, which is more prognostic [14]. Since cystic duct stump cancer is rarely found incidentally after a routine cholecystectomy for biliary colic, sampling of Calot's node is often missed due to either initial thermal injury or the lack of attention to lymph node sampling. This has some implications when it comes to staging of gallbladder carcinoma or when lymphadenectomy has to be performed, especially when at least 6 lymph nodes are recommended for lymphadenectomy [14]. Regardless, consideration of routine cystic lymph node biopsy may be prudent knowing the importance of cystic lymph node sampling.

To date, only a case report and a case series have identified low-grade cystic duct stump dysplasia after a nonmalignant cholecystectomy. Solaini et al. identified the first case of cystic duct stump dysplasia and only offered annual screening and monitoring [15]. The authors of the most recent case report pursued a cystic duct stump excision in light of prior findings of malignancy associated with high-grade cystic duct dysplasia. They however did not offer surveillance after excision [16]. Similarly, we pursued a laparoscopic cystic duct stump excision, without offering further surveillance or malignancy workup due to normal labs and the patient's excellent postoperative recovery. Our stump excision pathology showed reactive atypia without dysplasia, which was not unexpected given that it could be caused by specimen manipulation and chronic inflammation [17].

At the current state, management of low-grade cystic duct stump dysplasia after a routine laparoscopic cholecystectomy continues to be controversial. On the one hand, stump reexcision laparoscopically can be challenging technically and, as a result, unnecessarily exposes a patient to increased morbidity and mortality, should the risks of upgrading be negligible. On the other hand, if low-grade dysplasia poses a significant risk for upgrading, then early reexcision may be worth the extra morbidity. The other dilemma is whether surveillance should be offered. These decisions are difficult to make without adequate data. However, a multidisciplinary and holistic approach, taking into account a patient's comorbidities, risk of cancer given their genetic profile, family history, and baseline surgical morbidity, can help us make more informed decisions in managing these difficult clinical scenarios. As such, longitudinal observation studies are needed to determine the likelihood of low-grade dysplasia developing into a more advanced neoplastic process before we can offer a more well-informed guideline for managing such cases.

## References

[1] Jones M. W., Guay E., Deppen J. G. (2022). *Open Cholecystectomy*.

[2] Singh N., Zaidi A., Kaur R., Kaur J., Nijhawan V. S. (2022). Incidental gallbladder neoplasms: a growing global burden. *Cureus*.

[3] Rais R., Gonzalez I., Chatterjee D. (2019). Dysplasia in gallbladder: what should we do?. *Journal of Gastrointestinal Surgery*.

[4] Farrar D. A. (1951). Carcinoma of the cystic duct. *Journal of British Surgery*.

[5] Ozden I., Kamiya J., Nagino M. (2003). Cystic duct carcinoma: a proposal for a new “working definition”. *Langenbeck's Archives of Surgery*.

[6] Kim W. C., Lee D. H., Ahn S. I., Kim J. M. (2007). A case of cystic duct carcinoma treated with surgery and adjuvant radiotherapy: a proposal for new classification. *Journal of Gastrointestinal and Liver Diseases: JGLD*.

[7] Yokoyama Y., Nishio H., Ebata T. (2008). New classification of cystic duct carcinoma. *World Journal of Surgery*.

[8] Nakata T., Kobayashi A., Miwa S., Soeda J., Uehara T., Miyagawa S. (2009). Clinical and pathological features of primary carcinoma of the cystic duct. *Journal of Hepato-Biliary-Pancreatic Surgery.*.

[9] Nan L., Wang C., Dai Y. (2021). Cystic duct carcinoma: a new classification system and the clinicopathological features of 62 patients. *Frontiers in Oncology*.

[10] Bains L., Kaur D., Kakar A. K., Batish A., Rao S. (2017). Primary carcinoma of the cystic duct: a case report and review of classifications. *World Journal of Surgical Oncology*.

[11] Bickenbach K. A., Shia J., Klimstra D. S. (2011). High-grade dysplasia of the cystic duct margin in the absence of malignancy after cholecystectomy. *HPB*.

[12] Gavriilidis P., Askari A., Azoulay D. (2017). To resect or not to resect extrahepatic bile duct in gallbladder cancer?. *Journal of Clinical Medical Research*.

[13] Kokudo N., Makuuchi M., Natori T. (2003). Strategies for surgical treatment of gallbladder carcinoma based on information available before resection. *Archives of Surgery*.

[14] Sung Y. N., Song M., Lee J. H. (2020). Validation of the 8th edition of the American Joint Committee on Cancer staging system for gallbladder cancer and implications for the follow-up of patients without node dissection. *Cancer Research and Treatment*.

[15] Solaini L., Sharma A., Watt J., Iosifidou S., Chin Aleong J. A., Kocher H. M. (2014). Predictive factors for incidental gallbladder dysplasia and carcinoma. *The Journal of Surgical Research*.

[16] Cianfarani A., Mongelli F., Giuseppe M. D. (2019). Dysplasia of cystic duct and positive resection margins after cholecystectomy: a challenging decision making process. *Journal of Surgery*.

[17] Wrenn S. M., Callas P. W., Abu-Jaish W. (2017). Histopathological examination of specimen following cholecystectomy: are we accepting resect and discard?. *Surgical Endoscopy*.

